# Impact of Self-Reported Sitting Time and Transtheoretical Model Based on Exercise Behavior Change on Glycemic and Weight Control in Japanese Adults with Type 1 Diabetes: A Cross-Sectional Study

**DOI:** 10.3390/healthcare8020105

**Published:** 2020-04-22

**Authors:** Hiroto Honda, Makoto Igaki, Shin-ichiro Tanaka, Kumiko Ono, Yushi Hirota

**Affiliations:** 1 Department of Physical Therapy, Aino University, Ibaraki 567-0012, Japan; 2 Department of Rehabilitation, Toyooka Hospital Hidaka Medical Center, Toyooka 669-5392, Japan; 3 Department of Internal Medicine, Toyooka Hospital Hidaka Medical Center, Toyooka 669-5392, Japan; 4 Graduate School of Health Sciences, Kobe University, Kobe 654-0142, Japan; 5 Division of Diabetes and Endocrinology, The Department of Internal Medicine, Kobe University Graduate School of Medicine, Kobe 650-0017, Japan

**Keywords:** sitting time, transtheoretical model, self-administered questionnaire, glycemic control, weight control, type 1 diabetes

## Abstract

This cross-sectional study aimed to examine the associations among self-reported sitting time (ST), transtheoretical model (TTM) based on exercise behavior change, and glycemic and weight control in Japanese adults with type 1 diabetes (T1D). Forty-two adults (age, 44.0 (33.3–56.8) years) with uncomplicated T1D answered questions regarding their lifestyles, including ST per day, and TTM using self-administered questionnaires. The glycated hemoglobin (HbA1c) level correlated with age and ST (*p* < 0.05, *p* < 0.01, respectively), whereas body mass index correlated with duration of T1D and TTM (*p* < 0.05, *p* < 0.01, respectively). Logistic regression analysis showed that poor glycemic control (HbA1c, >7%) was associated with ST (odds ratio, 3.53 (95% confidence interval, 1.54–8.11), *p* < 0.01). In addition, the cut-off points for quartiles of ST were 4.6, 6.0, and 8.0 h/day, and the HbA1c level in the lowest quartile was 15% lower than that in the highest quartile (*p* < 0.01). Although further studies with larger samples are needed, these results implied that expanded self-reported ST might be related to poor glycemic control in Japanese T1D adults, most of whom were lean, young and middle-aged, regardless of TTM based on exercise behavior change.

## 1. Introduction

Sedentary behavior, which is mostly the same as sitting time (ST) or reclining, is a primary indication for the decrease of physical activity (PA) [1]. There are associations between sedentary behavior and increased risk for at least 35 chronic diseases and clinical conditions [2]. Furthermore, even if individuals perform exercise or moderate- to vigorous-intensity PA regularly, increased ST induces the incidences of cardiovascular diseases and mortality [3,4]. As for metabolic function, sedentary behavior causes the deterioration of glycemic control [5] and an increased risk of type 2 diabetes (T2D) [6]. Previous research showed that an increase in the amount of time spent in daily sedentary behavior caused poor glycemic control in T2D patients [7]. On the other hand, in individuals with type 1 diabetes (T1D), the association between sedentary behavior and health outcomes is unclear. Some studies investigated the association between these factors in children, adolescents, young adults, and adults of all ages with T1D, and they analyzed the presence [8,9,10] or absence [11,12] of any significant relationships. The American Diabetes Association guidelines state that the specific role of PA in the management of blood glucose (BG) and the prevention of diabetes complications for people with T1D is not as clear as it is for those with T2D [13].

Furthermore, there is no known study on the relationship between sedentary behavior and glycemic control in Japanese adults with T1D. The prevalence of T1D in Japan is lower than that in Western countries [14,15,16]. Management of T1D is developing at a fast pace through various devices, medication, and nutrition therapy, and Japanese individuals with T1D receive various treatments in accordance with their dietary habits, culture, residential environment, and physical status, which differ from those of other countries. Thus, it is uncertain whether results from Western patients [8,9,10,11,12] can be applied to Japanese individuals with T1D.

To assess and promote PA, the transtheoretical model (TTM), which is the belief in one’s ability to achieve certain goals [17], has been extensively used [18,19]. The constructs of this model include stages of change, change processes, decisional balance, and self-efficacy. TTM based on exercise behavior change is an important factor for diabetic patients performing exercise. In individuals with T2D, the progression across the TTM stages is associated with better health outcomes such as glycemic control [20,21]. However, little is known about how TTM contributes to the management of health outcomes in individuals with T1D.

Therefore, we conducted this study to examine the association among ST, TTM based on exercise behavior change, and glycemic and weight control in Japanese adults with T1D.

## 2. Materials and Methods

### 2.1. Study Design

This was a cross-sectional study. All participants provided written informed consent. The protocol for this study was approved by the institutional review board of Toyooka Hospital Hidaka Medical Center (Toyooka, Japan; approval number: 13, 2014) in accordance with the Declaration of Helsinki.

### 2.2. Participants

A total of 57 Japanese adults with T1D whose fasting serum C-peptide levels were <0.2 nmol/L and who regularly visited Toyooka Hospital Hidaka Medical Center (Toyooka, Japan) and Kobe University Hospital (Kobe, Japan) from 2015 to 2017 volunteered for this study. They usually visited the hospital once every 1–2 months as outpatients. T1D patients who were >20 years of age (no upper age limit) and were diagnosed over a year ago were included in the study. Patients with macrovascular or microvascular complications, motor dysfunctions, cognitive impairment, or other diseases affecting PA were excluded. All participants were being treated with insulin therapy, that is, multiple daily insulin injections or continuous subcutaneous insulin infusion (CSII), but no oral hypoglycemic agents. They also received medical nutrition therapy (based on carbohydrate counting) and self-management education for T1D, but no regular exercise therapy; it did not matter whether they performed exercise by themselves. They had no change in these treatments for at least 3 months.

### 2.3. Measurements

All variables, including glycated hemoglobin (HbA1c) determined using a high-performance liquid chromatographic method and body mass index (BMI), were measured when the participants visited the hospital as regular outpatients. On the same day, they were asked to answer questions regarding their lifestyles, including ST per day, use of CSII, habits of cigarette smoking (≥1 time/day) and alcohol drinking (≥1 day/week, alcohol ≥ 20 g/day), use of car, whether employed, living with family, and TTM based on exercise behavior change using the self-administered questionnaires.

The assessment of ST was based on a question from the International Physical Activity Questionnaire (IPAQ) [1], which is the most widely used PA questionnaire [22], and the validity of the Japanese version IPAQ was proved [23]; “During the last 7 days, how much time did you spend sitting on a weekday?” [1]. ST included time spent sitting or lying down, such as watching television, playing video games, computer usage, reading, deskwork, and sitting on a train or in a car.

The stages of exercise behavior change using the TTM were classified into five stages: precontemplation (not intending to take action in the foreseeable future, usually considered as the next six months), contemplation (intending to change in the next six months), preparation (intending to take action in the immediate future, usually considered as the next month), action (making specific overt modifications in lifestyles within the past six months), and maintenance (making specific overt modifications in lifestyles and working to prevent relapse) [17,24]. The TTM method in this study consisted of one question on the five stages mentioned above, the reliability and validity of which were proved for applicability in the Japanese population [25,26]. The participants answered using a dichotomous scale (yes/no). For example, they chose “I currently do not exercise, but I intend to start exercising in the next 6 months” if they were in the contemplation stage [25].

### 2.4. Statistical Analysis

All values are reported as the median (quartiles 25–75). All parameters were initially analyzed for the Shapiro–Wilk normality test to confirm a normal distribution. Correlations among HbA1c, BMI, and other variables were analyzed using Spearman’s rank correlation coefficient. Comparisons of variables in the groups categorized according to HbA1c (≤7% or >7% (poor glycemic control [27,28])) were analyzed using the Mann–Whitney U-test for continuous variables and Fisher’s exact test for nominal variables. Post hoc effect size and power analyses were performed for each variable, given the sample size. Binomial logistic regression analysis was performed to determine the predictors of poor HbA1c control, retraining variables with *p*-values < 0.1 in the above analyses as the explanatory variables. For a visual representation, quartiles of ST were calculated, and the Kruskal–Wallis test followed by the Steel–Dwass test were used to analyze differences in the HbA1c levels between the quartiles of ST. The results were analyzed using the IBM SPSS statistics (version 20.0, IBM, Tokyo, Japan). Statistical significance was set at *p* < 0.05.

## 3. Results

**Figure 1:**
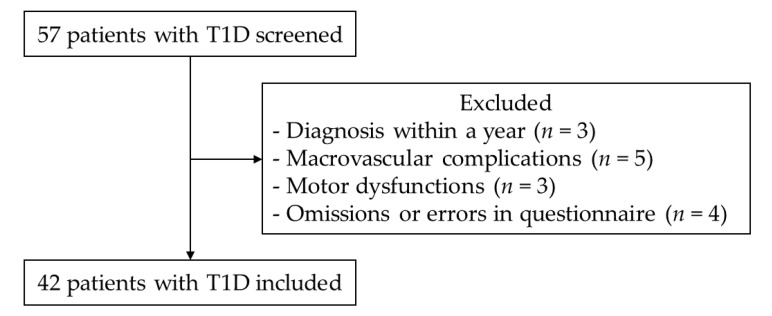
Patient screening flowchart. T1D: type 1 diabetes.

The patient screening flowchart is shown in Figure 1. Of the 57 T1D patients who were screened, 42 (age 24–74 years) were included in the analysis. The characteristics of the study participants are shown in Table 1. Of all the patients, 26 participants (61.9%) had poor glycemic control (HbA1c, >7.0%), and 2 participants (4.8%) had poor weight control (BMI, >25.0 kg/m^2^).

HbA1c had correlations with age and ST (*p* < 0.05, *p* < 0.01, respectively), whereas BMI had correlations with the duration of T1D and TTM (*p* < 0.05, *p* < 0.01, respectively) (Table 2).

In the analysis of variables of the groups with ≤7.0% or >7.0% of the HbA1c level, there were significant differences in ST and TTM (*p* < 0.01, *p* < 0.05, respectively) (Table 3).

For binomial logistic regression analysis, the HbA1c level (≤7.0% or >7.0%) was set as the objective variable, while BMI, ST, and TTM (with a *p*-value < 0.1 variables on comparisons between groups) were set as the explanatory variables (units: BMI, per 1-kg/m^2^ increase; ST, per 1-hour increase; TTM, per 1-stage increase). As a result, the HbA1c level was significantly associated only with ST (*p* < 0.01) (Table 4) after adjusting for age and gender. 

**Figure 2:**
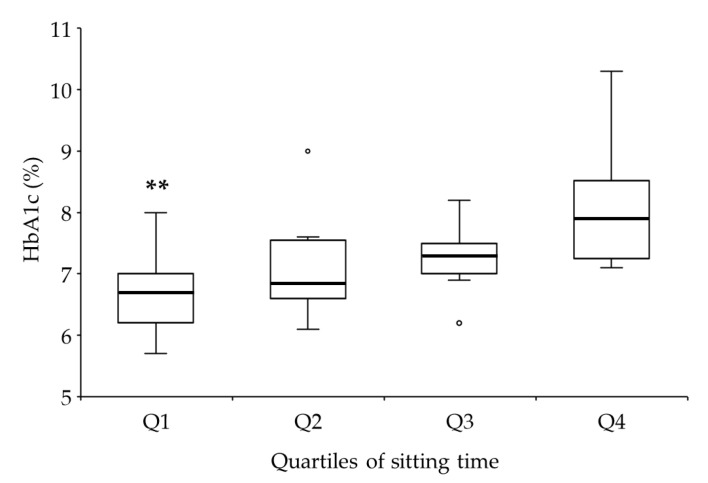
Quartiles of sitting time (ST) with glycated hemoglobin (HbA1c). The cut-off points for quartiles of ST were 4.6, 6.0, and 8.0 h/day. The black solid line indicates the median, the box represents the lower and upper quartiles, the upper and lower adjacent lines indicate the minimum and maximum values, and hollow circles represent outliers. ** *p* < 0.01 compared to quartile 4.

The cut-off points for quartiles of ST were 4.6, 6.0, and 8.0 h/day. The Kruskal–Wallis test showed a significant difference among quartiles (*p* < 0.01), and the post hoc analysis showed that the HbA1c level in the lowest quartile of ST was 15% lower than that in the highest quartile (*p* < 0.01) (Figure 2).

## 4. Discussion

To the best of our knowledge, this is the first study to elucidate the associations among ST, TTM based on exercise behavior change, and glycemic and weight control in Japanese adults with T1D. The major finding of the present study is that long ST has a relationship with high HbA1c level, independent of other lifestyles and TTM. Furthermore, time spent sitting for <4.6 h/day (the lowest quartile of ST) might be associated with good glycemic control.

PA is a physiologically relevant stimulus to promote glucose uptake in skeletal muscles, which is the largest glucose-utilizing organ in the human body. Although recommended PA for glycemic control should be of moderate- to vigorous-intensity and at least over 150 min/week [13], breaking sedentary behavior is also an important method for the management of BG profiles and it is required to be done frequently throughout the day [29,30,31]. The cumulative number of muscular contractions throughout a day during interruptions in sedentary time or low-intensity PA may require a greater energy demand than a single bout of continuous aerobic activity [32]. PA-induced glucose uptake in skeletal muscle is also intact in individuals with T1D [33]. Conversely, the lack of promoting PA or exercise may cause poor glycemic control, obesity, or macrovascular complications in individuals with T1D [8], although the evidence for this remains unclear [34]. Furthermore, physical inactivity can reduce fitness, such as VdotO_2_max [35] and muscle strength [36], which are important for performing PA. Therefore, we believe that long-time sedentary behavior and lack of strenuous PA resulted in a continuous reduction of glucose uptake and induced high HbA1c levels in this study population.

Japan is one of the countries with the longest ST; the average ST in Japanese adults was 7.0 h/day [37], investigated with the help of IPAQ in a cross-sectional study. A recent Japanese cross-sectional study that used IPAQ on 1053 patients with T2D showed that average ST was 6.0 h/day [38]. However, in T1D patients, little is known about average or median ST. The median ST in this study was 6.0 h/day, thus making the participants slightly more active than the general Japanese population and equally active as T2D patients. In the present study, there was a dose-response of ST for HbA1c; however, the second and third quartiles of ST, which included the median value (6.0 h/day), did not yield significantly better glycemic control than the highest quartile of ST (Figure 2). Conversely, the lowest quartile of ST, which was <4.6 h/day, was better than the highest quartile of ST for glycemic control. Thus, shorter ST than that of the general Japanese population may be necessary for good glycemic control in T1D patients.

As an important note about the interpretation of the result, ST based on the IPAQ inquiring last week’s lifestyle may not relate to current glycemic control because HbA1c reflects average BG levels over approximately 3 months [39]. However, PA measured by IPAQ may be correlated with that measured by an accelerator for the same period [40], although it is unclear whether PAs of the last 7 days and 3 months are equivalent. In addition, a previous study showed that the “last 7 days recall” method in IPAQ is similar to the “usual week recall” method [1]. Thus, we consider that ST measured by the “last 7 days recall” method in this study is acceptable, although it did not exactly correspond to actual ST value in the last 3 months.

In the present study, TTM based on exercise behavior change correlated with BMI but not with HbA1c. T1D patients have many barriers to PA, similar to the general public, such as weather conditions, lack of time, and the requirement for an exercise machine, in addition to fear of hypoglycemia, which may be the main PA barrier in people with T1D [41,42]. Insulin users are usually advised to consume carbohydrates to prevent hypoglycemia during and after PA. Glycemic control is influenced by the average BG level as well as its range of change [39], where bouts of aerobic, resistance, or sprint exercise widely reduces the BG level [43,44]; however, the range of BG levels during and after exercise may be high in T1D patients because they consume carbohydrates containing foods during hypoglycemic conditions [45]. Thus, although exercise is an important strategy for diabetes management, TTM, which has a correlation with exercise behavior [46], may have no relationship with HbA1c levels in T1D patients. On the other hand, weight control is affected by activity volume and energy expenditure [47]. Hence, the progression across TTM stages might be associated with BMI in the present study.

As a note, there is a similar caution with the assessment timing of ST, that HbA1c and BMI responses may not be affected by a current TTM stage, which includes the intention of exercise behavior within the next 6 months [17]. However, TTM stages also state either “I currently do not exercise” or “I currently exercise but not regularly” as the present exercise behavior. The progress of TTM is associated with a higher level of self-management and BMI at the same timing of measurement in T2D patients [48]. Therefore, we consider that the current TTM stage reflects exercise habits before a few months, although it did not exactly correspond to that in the last 3 months; HbA1c and BMI responses might be affected by TTM stages assessed in this study.

ST and TTM stages in this study may be good or bad for preventing mortality, metabolic disorders, frailty, chronic diseases, or other healthy behaviors. The median ST in this study was 6.0 h/day. Two meta-analyses revealed that ST of more than 7 h/day based on 6 studies (5 using self-reported measures) [49] or 4 h/day based on 13 studies (all using self-reported measures) [50] were associated with increased mortality risks. Conversely, a previous study using IPAQ showed that ST of more than 2 or 4 h/day induced abdominal obesity or high blood pressure [51]. In addition, the cut-off points for time spent sitting as an indicator of frailty were more than 4.3 h/day for males and more than 5.5 h/day for females [52]. Assessment and intervention using TTM are useful for the management of various chronic diseases, such as T2D, hypertension, osteoporosis, or cancer, and other habits such as eating, sleeping, or smoking [53]. In this study, 53.8% of patients with poor glycemic control were in the precontemplation stage of TTM. Thus, ST and TTM in this study may induce other unhealthy effects, although they may not increase mortality risks.

There are some limitations to this study. First, the sample size is small because of lack of facilities, and most participants were lean, young, and middle-aged adults. The small sample size is likely to cause type II errors in statistical analysis [54]. However, the post hoc power analysis in this study showed that there was enough power for analyzing the relationship between ST and HbA1c. Therefore, although this study had a small sample size, which might have resulted in a selection bias, we believe that the association observed in this study is appropriate. Based on this preliminary result, a larger sample size is needed to confirm the impact of ST on glycemic control in T1D patients with various weights and age ranges. Second, we recruited patients whose T1D were uncomplicated. Leisure-time PA of T1D patients with diabetic complications tended to be of lower frequency and lower intensity than that of patients without complications [55]. Thus, the results may not apply to complicated T1D patients. Third, ST was not evaluated objectively, such as by using a tri-axial accelerometer; hence, the ST measured subjectively in this study might lack accuracy. Although the validity in Japanese diabetic adults was confirmed [23], a recent study showed that the IPAQ might underestimate ST measured by an objective criterion due to recall bias [56]. As mentioned above, there might be variances between measured and actual HbA1c and ST values, as well as TTM stages, because HbA1c reflects average BG levels approximately 3 months ago and not present BG levels [39]. In addition, we did not include all the contents of IPAQ, as ST was the only main target of this study, and the type, intensity, duration, and timing of PA, including activities of work and leisure time, that affect glycemic control in Japanese adults with T1D are unclear. Furthermore, the influence of the percentage of ST in the total time of wakefulness also remains unclear because we did not log any sleep time. Thus, further studies are needed to confirm the clinical relevance of PA by objective methods, such as smart watches and other wearable accelerometers.

## 5. Conclusions

In summary, we found that self-reported ST was related to glycemic control, and TTM based on exercise behavior change was related to weight control in Japanese adults with T1D, most of whom were lean, young, and middle-aged. Furthermore, the association between ST and HbA1c was observed even during progression across the TTM stages. Although further studies with larger samples are needed, we propose that the assessment of ST is important for the management of T1D.

## Figures and Tables

**Table 1:** Characteristics of the study participants (*n* = 42).

Variables	Values
Male/Female (frequency) (percentage)	14/28 (33.3/66.7)
Age (years)	44.0 (33.3–56.8)
Duration of T1D (years)	11.0 (3.1–18.8)
BMI (kg/m^2^)	22.1 (20.9–23.2)
HbA1c (%)	7.2 (6.7–7.8)
Sitting time (hours/day)	6.0 (4.6–8.0)
Use of CSII (frequency) (percentage)	11 (26.2)
Cigarette smoking (frequency) (percentage)	10 (23.8)
Alcohol drinking (frequency) (percentage)	18 (42.9)
Use of car (frequency) (percentage)	21 (50.0)
Employed (frequency) (percentage)	26 (61.9)
Living with family (frequency) (percentage)	30 (71.4)
TTM based on exercise behavior change (frequency) (percentage)	
Precontemplation	18 (42.9)
Contemplation	3 (7.1)
Preparation	6 (14.3)
Action	1 (2.4)
Maintenance	14 (33.3)

T1D: type 1 diabetes; BMI: body mass index; HbA1c: glycated hemoglobin; CSII: continuous subcutaneous insulin infusion; TTM: transtheoretical model. Values of continuous variables are presented as the median (quartiles 25–75).

**Table 2:** Correlations among HbA1c, BMI, and other variables.

Variables	HbA1c			BMI		
	*r*	*p*-Value	Power	*r*	*p*-Value	Power
Gender	–0.20	0.21	0.25	0.14	0.39	0.14
Age	0.35	0.02 *	0.64	0.23	0.14	0.31
Duration of T1D	0.22	0.16	0.29	0.39	0.01 *	0.74
BMI	0.20	0.20	0.25	-	-	-
HbA1c	-	-	-	0.20	0.20	0.25
Sitting time	0.60	<0.01 **	0.99	<0.01	0.99	0.05
Use of CSII	–0.30	0.05	0.50	–0.07	0.65	0.07
Cigarette smoking	–0.23	0.15	0.31	0.10	0.54	0.10
Alcohol drinking	–0.27	0.08	0.42	0.03	0.83	0.05
Use of car	–0.03	0.87	0.05	–0.06	0.73	0.07
Employed	–0.19	0.22	0.23	0.09	0.56	0.09
Living with family	–0.27	0.08	0.42	–0.06	0.72	0.07
TTM	–0.26	0.09	0.39	–0.40	<0.01 **	0.76

HbA1c: glycated hemoglobin; BMI: body mass index; T1D: type 1 diabetes; CSII: continuous subcutaneous insulin infusion; TTM: transtheoretical model. * *p* < 0.05, ** *p* < 0.01.

**Table 3:** Comparisons between the groups with HbA1c ≤ 7.0% and > 7.0%.

Variables	HbA1c		*p*-Value	Effect Size (*r*, Cramer’s *V*)	Power
	≤7% (*n* = 16)	>7% (*n* = 26)			
Male/Female (frequency) (percentage)	7/9 (43.8/56.2)	7/19 (26.9/73.1)	0.32	0.12	0.11
Age (years)	38.5 (33.8–47.5)	45.5 (33.8–61.8)	0.22	0.50	0.32
Duration of T1D (years)	6.0 (2.0–18.3)	13.0 (5.5–18.8)	0.17	0.22	0.10
BMI (kg/m^2^)	21.4 (20.0–23.0)	22.8 (21.1–23.5)	0.09	0.63	0.48
Sitting time (hours/day)	4.0 (3.0–5.5)	7.3 (6.0–8.0)	<0.01 **	1.56	0.97
Use of CSII (frequency) (percentage)	6 (37.5)	5 (19.2)	0.28	0.15	0.15
Cigarette smoking (frequency) (percentage)	5 (31.3)	5 (19.2)	0.47	0.08	0.06
Alcohol drinking (frequency) (percentage)	9 (56.3)	9 (34.6)	0.21	0.16	0.17
Use of car (frequency) (percentage)	8 (50.0)	13 (50.0)	1.00	0.00	0.00
Employed (frequency) (percentage)	10 (62.5)	16 (61.5)	1.00	0.00	0.00
Living with family (frequency) (percentage)	13 (81.3)	17 (65.4)	0.32	0.12	0.08
TTM based on exercise behavior change (frequency) (percentage)			0.04 *	0.46	0.69
Precontemplation	4 (25.0)	14 (53.8)			
Contemplation	0 (0.0)	3 (11.5)			
Preparation	3 (18.8)	3 (11.5)			
Action	0 (0.0)	1 (3.8)			
Maintenance	9 (56.3)	5 (19.2)			

HbA1c: glycated hemoglobin; T1D: type 1 diabetes; BMI: body mass index; CSII: continuous subcutaneous insulin infusion; TTM: transtheoretical model. Values of continuous variables are presented as the median (quartiles 25–75). * *p* < 0.05, ** *p* < 0.01.

**Table 4:** Binomial logistic regression analysis for HbA1c (age-gender adjusted analysis).

Variables	Odds Ratio	95% CI	*p*-Value	VIF
BMI (per 1-kg/m^2^ increase)	1.52	0.86–2.69	0.15	1.18
Sitting time (per 1-hour increase)	3.53	1.54–8.11	<0.01 **	1.55
TTM based on exercise behavior change (per 1-stage increase)	0.52	0.27–1.01	0.07	1.43

AIC (Akaike’s information criterion) = 33.81. HbA1c: glycated hemoglobin; CI: confidence interval; VIF: variance inflation factor; BMI: body mass index; TTM: transtheoretical model. ** *p* < 0.01.
